# Population Pharmacokinetics of Intravenous Amoxicillin Combined With Clavulanic Acid in Healthy and Critically Ill Dogs

**DOI:** 10.3389/fvets.2021.770202

**Published:** 2021-11-15

**Authors:** Maria D. Vegas Cómitre, Stefano Cortellini, Marc Cherlet, Mathias Devreese, Beatrice B. Roques, Alain Bousquet-Melou, Pierre-Louis Toutain, Ludovic Pelligand

**Affiliations:** ^1^Department of Clinical Science and Services, The Royal Veterinary College, Hatfield, United Kingdom; ^2^Department of Pharmacology, Toxicology and Biochemistry, Faculty of Veterinary Medicine, Ghent University, Merelbeke, Belgium; ^3^INTHERES, Université de Toulouse, INRAE, ENVT, Toulouse, France; ^4^Department of Comparative Biomedical Science, The Royal Veterinary College, Hatfield, United Kingdom

**Keywords:** antimicrobial, MIC, clinical breakpoint, Augmentin, ICU, VetCast, PK/PD, NLME

## Abstract

**Background:** Data regarding antimicrobial pharmacokinetics (PK) in critically ill dogs are lacking and likely differ from those of healthy dogs. The aim of this work is to describe a population PK model for intravenous (IV) amoxicillin–clavulanic acid (AMC) in both healthy and sick dogs and to simulate a range of clinical dosing scenarios to compute PK/PD cutoffs for both populations.

**Methods:** This study used a prospective clinical trial in normal and critically ill dogs. Twelve client-owned dogs hospitalized in the intensive care unit (ICU) received IV AMC 20 mg/kg every 8 h (0.5-h infusion) during at least 48 h. Eight blood samples were collected at predetermined times, including four trough samples before the next administration. Clinical covariates and outcome were recorded, including survival to discharge and bacteriologic clinical failure. Satellite PK data were obtained *de novo* from a group of 12 healthy research dogs that were dosed with a single AMC 20 mg/kg IV. Non-linear mixed-effects model was used to estimate the PK parameters (and the effect of health upon them) together with variability within and between subjects. Monte Carlo simulations were performed with seven dosage regimens (standard and increased doses). The correlation between model-derived drug exposure and clinical covariates was tested with Spearman's non-parametric correlation analysis. Outcome was recorded including survival to discharge and bacteriologic clinical failure.

**Results:** A total of 218 amoxicillin concentrations in plasma were available for healthy and sick dogs. A tricompartmental model best described the data. Amoxicillin clearance was reduced by 56% in sick dogs (0.147 L/kg/h) compared with healthy dogs (0.336 L/kg/h); intercompartmental clearance was also decreased (*p* <0.01). None of the clinical data covariates were significantly correlated with individual exposure. Monte Carlo simulations showed that higher PK/PD cutoff values of 8 mg/L could be reached in sick dogs by extending the infusion to 3 h or doubling the dose.

**Conclusions:** The PK of AMC is profoundly different in critically ill dogs compared with normal dogs, with much higher interindividual variability and a lower systemic clearance. Our study allows to generate hypotheses with regard to higher AMC exposure in clinical dogs and provides supporting data to revise current AMC clinical breakpoint for IV administration.

## Introduction

Amoxicillin and amoxicillin in combination with clavulanic acid (AMC) are the most frequently used antimicrobials worldwide according to the World Health Organization (1) and the most prescribed antimicrobial in veterinary medicine, including companion animals (2). In companion animals, AMC combinations have been historically licensed against Gram positives and Gram negatives, including *Escherichia coli*. These include veterinary licensed products (oral tablets and injectable suspensions) and human products as solutions for intravenous (IV) administration. The extent of use of the IV formulation in the veterinary perioperative and critical care contexts is high in Europe, but exact figures are not available (3).

Nowadays, clinical breakpoints (CBPs) for interpretation of antimicrobial susceptibility testing (AST) are determined for bacterial targets based on a combination of three values for each animal species (4, 5). The first one is the highest concentration included in the minimum inhibitory concentration (MIC) distribution of the wild-type population, designed as Epidemiological Cut-Off (ECOFF). The second one is the pharmacokinetic/pharmacodynamic cutoff (PK/PD_CO_) (6), which is determined using a population PK model developed for the species in question. In practice, Monte Carlo simulations are used to compute PK/PD_CO_ as the highest MIC for which the target value of the PK/PD efficiency index is actually achieved in 90% of subjects. PK/PD index includes either the percentage of time for which free plasma concentration exceeds MIC within the dosing interval (%fT > MIC, typically used for beta lactams) or a given average level of exposure for fAUC/MIC (i.e., how many MIC-fold the average free plasma concentration is). Finally, the clinical cutoff is the highest MIC associated with clinical cure, but currently no data are available in veterinary medicine; PK/PD_CO_ consequently retains a pivotal role in the establishment of veterinary CBP.

Human CBP values are established by national or international organizations such as the European Committee on Antimicrobial Susceptibility Testing (EUCAST) or Clinical and Laboratory Standards Institute (CLSI). These organizations typically publish three values to interpret AST results as susceptible (S), intermediary (I), or resistant (R). They are established for some typical dosage regimens. For a systemic effect, EUCAST reports a single value of 8 mg/L for amoxicillin (7) to classify *E. coli* strains either as susceptible (S ≤ 8 mg/L) or resistant (R > 8 mg/L). The VAST/CLSI, which is the veterinary section of CLSI, does not directly test the sensitivity to amoxicillin but relates to ampicillin as a class representative. For skin and soft tissue infection in dogs, two CBP are given for *Staphylococcus pseudintermedius*, ≤ 0.25 mg/L for S and >0.5 mg/L for R, whereas a single CBP of ≤ 0.25 mg/L is given for *Streptococcus canis*, based on an oral amoxicillin regimen of 11 mg/kg at 12-h interval. For *E. coli* in canine skin and soft tissue infections, the three CBPs are ≤ 0.25 (S), 0.5 (I), and ≥ 1 mg/L (R) for the same dosage oral regimen (8). It should be stressed that CBP for R ≥ 1 mg/L virtually precludes the use of IV amoxicillin against systemic infections with species whose ECOFF is 8 mg/L (as *E. coli*) (9).

Many studies in human patients receiving antimicrobials in the critical care setting demonstrated that drug exposures are often inadequate in critical patients due to the alterations in PK parameters associated with several severe conditions (10–13). This has repercussions on the interpretation of CBPs, which have been established with kinetics obtained from healthy patients in human medicine and most often on healthy animals in veterinary medicine in an experimental setting (i.e., often not representative of clinical conditions in terms of breed, age, co-medications). This highlights how fundamental is the understanding of PK of specific antimicrobials in diseased patients when considering CBP, dose, and administration intervals. We hypothesized differences in beta-lactam exposure between the two populations, i.e., sick and healthy dogs, and high variability between and possibly within sick hospitalized dogs with comorbidities (14, 15).

The goals of the present study were to (i) elaborate a population PK model for IV amoxicillin in 12 healthy dogs and 12 critically ill dogs for which demographics, hospitalization variables, and bacteriological and clinical outcomes are reported; (ii) relate model-derived drug exposure with aforementioned variables and outcomes; and (iii) simulate a range of clinical doses and dosing scenario (intermittent administration vs. extended or continuous infusions) to compute and contrast the PK/PD_CO_ for both populations. These PK/PD_CO_ values could be used subsequently to revise the CBP for IV AMC use.

## Materials and Methods

Studies on sick and healthy dogs were performed in parallel and coordinated by the same investigator. Both studies were performed under the European Directive 2010/63/EU on the protection of animals used for scientific purposes [Animal (Scientific Procedures) Act in the United Kingdom]. For the Royal Veterinary College (RVC) dogs, the study protocol was approved by the Animal Welfare and Ethical Review Board. The satellite study was authorized by the French Ministry of Research and approved by the ethical committee for pharmacology toxicology Midi-Pyrénées (no. 86).

### Study Design for Sick Dogs

This prospective population PK study involving client-owned dogs was carried out at a University teaching hospital. Dogs hospitalized in the intensive care unit (ICU) with prescribed IV AMC acid (Augmentin® 1.2 g for reconstitution as an IV injection or infusion: 1 g of amoxicillin as amoxicillin sodium and 200 mg of clavulanic acid as potassium clavulanate; GlaxoSmithKline UK, Brentford, UK) and with a sampling line placed, such as a long stay or central venous catheter, placed for clinical reasons only, were eligible for the study. Dogs that had already received IV AMC prior to study enrolment were eligible for inclusion, as long as previous doses and administration times were exactly known. Exclusion criteria were dogs with body weight of <10 kg, packed cell volume of <20%, and previous oral or subcutaneous administration of AMC within the previous 2 and 7 days respectively.

### Drug Administration and Blood Sampling in Sick Dogs

Once enrolled, clinical dogs were administered 20 mg/kg of AMC (corresponding to 16.667 mg/kg of amoxicillin) intravenously as an infusion over 30 min, started at time T+0 min. Doses were given every 8 h during a minimum period of 48 h, using a calibrated syringe driver. The decision on antimicrobial therapy and the placement of long stay catheter was ultimately dependent on the primary clinician only. The animals would be withdrawn from the study if the antimicrobial was discontinued or changed to a different antimicrobial or formulation, or if no sampling line was placed.

Blood samples (1.3 mL/sample) were collected prior to antimicrobial administration (T0) if the dog had already received an IV AMC dose. All dogs already on AMC received the last dose at least 8 h prior to starting sampling. The following samples were collected 30 min later (T+30 min) at the end of the infusion then at 1, 2, and 4 h, and trough samples were collected at 8, 24, and 48 h. Blood samples were collected in EDTA tube and centrifuged for 10 min at 3,000 *g* immediately after withdrawal. Following centrifugation, the separated plasma was frozen at −80°C within 30 min from collection and processed within a maximum of 1 year.

### Pharmacokinetics in Healthy Beagle Dogs

Raw data from older studies reporting IV amoxicillin PK in laboratory dogs (16) were unavailable for computation of PK/PD_CO_ with the present modern tools. Therefore, to contrast sick and healthy dogs, we generated satellite PK-rich data sets from a group of 12 healthy laboratory beagles (Table 1). The healthy dog population was 12 female intact beagles. Median age was 24 months. They were dosed with 20 mg/kg IV bolus of AMC (no infusion) at the Toulouse Veterinary School. Blood sampling (2 mL/sample) was performed prior to administration and 2, 10, 25, 40 min, 1, 2, 4, 6, 8, 10, and 12 h after AMC administration. Blood samples were collected in lithium vs EDTA heparin tube and centrifuged at +4°C for 10 min at 3,000 g. Following centrifugation, the separated plasma was frozen at −80°C until further analysis.

**Table 1:** Demographics and experimental setup for the RVC clinical study (*n* = 12 dogs) and the satellite study (laboratory, *n* = 12 ENVT dogs).

**Subjects**	**Weight average (range) (kg)**	**Age average (range) (months)**	**Status**	**Breed**	**Formulation/route**	**Doses amoxicillin (mg/kg) average (±SD)**	**Design**	**Blood sampling schedule (h)**	**Analytical range for amoxicillin**
12 intact female beagles	11.5 (9.9 to 13.2)	24	Healthy	Beagle	Augmentin®/IV (bolus)	16.95 (±0.37)	Single dose, experimental	0, 0.03, 0.17, 0.42, 0.67, 1, 2, 4, 6, 8, 10, 12	50–25,000 ng/mL
									
									
12 mixed breeds*	20.6 (11.4 to 42.0)	40.2 (range 4.8 to 114)	Diseased	Mixed breed	Augmentin®/IV (0.5-h infusion)	16.66	Repeated dose, clinical	0, end of infusion (0.5), 1, 2, 4 and trough samples 8, 24, 48	50–25,000 ng/mL

^*^12 dogs recruited at Royal Veterinary College (RVC) and presented in Table 2.

### Biochemical Assays and Quantification of Amoxicillin Using Liquid Chromatography–Tandem Mass Spectrometry

Admission serum creatinine and albumin concentration were measured with a calibrated Beckman Coulter (Brea, CA, USA) AU680 biochemistry analyzer. Plasma amoxicillin concentrations of sick and healthy dogs were analyzed by the same laboratory, using ultra-high-performance liquid chromatography coupled to tandem mass spectrometry (UPLC-MS/MS), after a deproteinization step with acetonitrile, and a back-extraction of the acetonitrile with dichloromethane, as previously reported (17). In-life validation was conducted according to analytical methodology standards stated in Commission Decision 2002/657/EC (18).

Amoxicillin protein binding was measured in clinical dogs (plasma samples obtained from an independent group of eight dogs hospitalized in the ICU and receiving amoxicillin) and in one healthy dog (plasma sample spiked at three known amoxicillin concentrations and incubated *ex vivo* for 1 h at 38.5°C). Plasma samples were split into two aliquots: one was pH-buffered and ultrafiltrated (Pall Nanosep Omega 10K ultrafiltration device (ref. OD010C33), Pall Corporation, Portsmouth, UK) at 25°C for a minimum of 40 min (1,500 *g*), and the other one was kept in the same conditions but not centrifuged, as a temperature control. Both plasma and ultrafiltrate were frozen at ≤ -70°C until analysis. Stability for both drugs was demonstrated for at least 15 months at this temperature.

### Clinical Covariate Data and Outcome Assessment

We collected data from expected covariates including demographics (age, breed, and weight), disease diagnosis, and severity assessed by the acute patient physiologic and laboratory evaluation (APPLE_FAST_) score, systemic inflammatory response syndrome (SIRS) criteria (19), presence of infection, use and type of vasopressors, presence of surgical disease, institution of mechanical ventilation, fluid therapy administration (mL/kg/h), and acute kidney injury (AKI) grade (20). All covariate values were assessed during the sampling period only.

We recorded the outcome including survival to discharge, the recurrence of the disease within the hospitalization, and clinical failure. Clinical failure was defined as the requirement of adding a second antimicrobial drug based on initial culture results or clinical deterioration requiring escalation of antibiosis.

### Pharmacokinetic Analysis

Amoxicillin PK was evaluated using non-linear mixed-effects model (Phoenix NLME, version 8.3, Certara, Princeton, NJ, USA), which estimates the PK parameters and the variability (both between and within subject). Two and three compartment models were tested to fit plasma amoxicillin concentrations of the two studies.

Between-subject variability (BSV) for a parameter P was described using an exponential model, expressed as follows (6):


(1) $$\begin{array}{l} P_{i}\;=\;tvP{\;}^{*}\;exp\left(\eta P_{i}\right) \end{array}$$

where *tvP* is the typical value of the parameter within the population, and the random parameter η*P*_*i*_ (*eta*) represents the deviation from the typical value for the *i*th individual. *Etas* are assumed normally distributed with a mean of 0 and a variance of ω^2^. BSV was expressed as coefficients of variation with the following equation:


(2) $$\begin{array}{l} CV\left(\%\right)=100\times \sqrt{\;exp\left(\omega^{2}\right)-1} \end{array}$$

For model evaluation, a significant decrease in the Bayesian information criterion (BIC) as well as observation of observed vs. population and individual predicted concentrations plots were utilized. We used the M3 method (21) to handle data below the quantification limit (BQL), which enables estimation of the likelihood BQL measurement being real BQL data. Residual variability was described with a combination of additive and proportional error model. Diagonal vs. full variance–covariance matrices were compared, and full matrix was used for subsequent Monte Carlo simulations.

To evaluate the effect of covariate Health and Occasion, a stepwise covariate search was carried out onto selected PK parameters, with a BIC threshold of >6.63 points for forward inclusion and >10.83 points for backwards elimination.

Between-occasion variability (BOV; i.e., within subject) was tested on clearance (Cl). Four troughs were measured in the RVC dataset and were coded as different occasions (for doses before and up to first PK point = Occasion 1; for doses including the PK period with its 8 h trough and next dose = Occasion 2; for doses including the 24 h trough and next dose = Occasion 3; and for doses including 48 h trough and onwards = Occasion 4).

The final population model was evaluated using a non-parametric bootstrap sampling procedure (*n* = 30) and a Visual Predictive Check (VPC; *n* = 300) that compares observed with predicted quantiles.

### Assessment of Dose–Exposure Relationship and Computation of Pharmacokinetic/Pharmacodynamic Cutoff

Monte Carlo simulation (*n* = 1,000 patients) were performed with each of the seven following dosage regimens for the two populations (sick and healthy dogs): (i) standard regimen (20 mg/kg (16.667 mg/kg of amoxicillin) every 8 h, given in 0.5 h), (ii) extended/continuous infusions (20 mg/kg q 8 h, given in extended infusions lasting 1, 2, or 3 h or as an 8-h continuous infusion), or (iii) increased doses (20 mg/kg q 4 h, given in 0.5 h, or 40 mg/kg q 8 h, given in 0.5 h).

Individual plasma concentration time curves were generated for up to 96 h. BOV was included for the simulation of plasma concentration–time curves for the simulation in sick dogs (Occasion 1, 0–24 h; Occasion 2, 24–48 h; Occasion 3, 48–72 h; and Occasion 4, 72–96 h) but not for the healthy dogs. The fraction of time during which the free concentration exceeded the MIC (%fT > MIC) was calculated over the course of a 72-h treatment. PK/PD exposure target was defined at 40% fT > MIC. The PK/PD_CO_ is the highest MIC for which 90% of the simulated population achieves the PK/PD target (5). A target MIC of 8 mg/L was chosen at the worst-case scenario to cover the wild-type distribution of *E. coli* (EUCAST ECOFF: 8 mg/L).

### Prediction of Exposure by Clinical Variables

All sick dogs received a minimum of two administrations (16-h exposure) prior to the 48-h PK period. The correlation between model-derived area under the curve (AUC_0−64*h*_) and clinical covariates (APPLE_FAST_, albumin, creatinine, requirement for vasopressors, fluid therapy rate (mL/kg/h), and AKI grade score) was tested with Spearman's non-parametric correlation analysis (*p* < 0.05 was considered significant).

## Results

### Study Population

Sixteen dogs were recruited at the RVC between July 2019 and January 2021. Four dogs were excluded before any sample could be obtained due to jugular catheter malfunction (one), accidental removal of the jugular catheter (one), euthanasia following admission (one), and transition onto oral antimicrobial during the study period (one).

The demographics of the population of healthy dogs from Toulouse are reported in Table 1, and the demographics and covariates of sick dogs from RVC are reported in Tables 1–3.

**Table 2:** Demographic, clinical, and treatment characteristics of sick dog population.

**Characteristics**	**Percentage % (number dogs)**
Breed	
English springer spaniel	25 (3)
Labrador retrievers	25 (3)
Cocker Spaniel	8 (1)
Irish terrier	8 (1)
Japanese Akita	8 (1)
Boerboel	8 (1)
Bullmastiff	8 (1)
Cross-breed	8 (1)
Sex	
Male	33 (4)
Male neutered	50 (6)
Female	8 (1)
Female spayed	8 (1)
Primary disease	
•**Septic peritonitis**^**a**^	**58 (7)**
°Dehiscence from previous GI surgery	16 (2)
°Sub-lumbar abscess	16 (2)
°NSAID-related GI perforation	16 (2)
°Idiopathic peritonitis	8 (1)
•**Pyothorax**^**a**^	**25 (3)**
°Gastric penetrating foreign body into thorax	8 (1)
°Unknown etiology	16 (2)
°Parapneumonic effusion	8 (1)
•**Acute hemorrhagic diarrhea syndrome**	**8 (1)**
•**Burns (2nd and 3rd degrees)**	**8 (1)**
AKI^b, c^	16 (2)
Mechanical ventilation^b^	8 (1)

*GI, gastrointestinal; NSAIDs, non-steroidal anti-inflammatory drugs; AKI, acute kidney injury.*

*^a^All dogs with septic peritonitis and pyothorax had surgery in the 24 h prior to enrolment to the study.*

*^b^During sampling period.*

*^c^AKI IRIS grading 2 in both dogs*.

**Table 3:** Clinical and laboratory covariates for the sick dog population.

**Covariates**	**Value [median (25–75% interquartile range)]**	**Reference range and units**
${\text{APPLE}}_{\text{fast}}^{\text{†}}$	22.5 [IQR 20.25–29.75]	0–50
Albumin	21.6 [IQR 16.2–23.3]	26.3–38.2 g/L
Creatinine	62.5 [IQR 39.0–132.5]	20–144.5 μmol/L
Crystalloid volume	3 [IQR 2.50–3.55]	(mL/kg/h)
Vasopressors^§^	3	

*Acute patient physiologic and laboratory evaluation.*

*^§^Number of patients receiving vasopressors during the sampling period up to 12, 24, and 48 h*.

There were 91% (11/12) dogs that met SIRS criteria on admission (19). One dog with septic peritonitis did not fulfill the criteria. In addition, 10/12 dogs (83%) had a confirmed infectious focus identified. Although the dog presenting with acute hemorrhagic diarrhea syndrome (AHDS) did not have a confirmed source of infection, he had clinical signs compatible with septic shock, including tachycardia, tachypnea, and severe hypotension not responsive to fluid therapy or vasopressors, and was ultimately euthanized due to clinical deterioration. One dog suffered burns with 30% of his body surface affected but did not develop signs of infection.

Pathogens were grown in 58% (7/12) of dogs and in 70% (7/10) of dogs treated for infection with culture results available. Clinical failure rate was 41% (5/12) requiring an additional antimicrobial drug. However, 25% of dogs had a relapsing septic peritonitis: relapse of sub-lumbar abscessation (one) and dehiscence of enterectomy or enterotomy (two). The pathogens identified in dogs with relapse of septic peritonitis were *E. coli* (two), anaerobes (two), *Pasteurella stomatis* (one), alpha-hemolytic *Streptococcus* spp. (one), and *Enterococcus faecalis* (one). Multidrug-resistant (MDR) pathogen *Klebsiella pneumoniae* was isolated from one dog with pyothorax and pneumonia requiring mechanical ventilation with subsequent antimicrobial escalation. One dog with relapse septic peritonitis with a primary culture of *E. coli* sensitive to AMC grew *MDR E. coli* and MDR *mucoid E. coli* on his second culture following dehiscence and was ultimately euthanized after relapsing for a third time. No pathogen could be identified in two dogs with septic peritonitis.

Nine dogs (75%) survived to discharge; the remaining three dogs were euthanized based on perceived poor prognosis and welfare grounds. One dog with AHDS was euthanized due to acute deterioration, and two dogs with septic peritonitis had a relapse of their abdominal infection.

### Population Pharmacokinetic Model

A total of 218 amoxicillin concentrations in plasma were available for the healthy and sick dogs. The limit of quantification of the analytical method was 50 ng/mL. Within-days (between-days) accuracy and precision were lower than 6.3% (6.2%) and 2.5% (3.6%), respectively, and therefore fell within the acceptance criteria.

A three-compartment model for amoxicillin best described the data, including six PK parameters (Cl total clearance; V1 central volume of distributions; volumes of the superficial and deep distribution compartments, V2 and V3, respectively; and associated intercompartmental clearances Cl2 and Cl3). BSV was modeled for Cl, Cl2, V1, and V2. Covariate search demonstrated significant reduction in Cl and Cl2 for the diseased state. BOV was supported for Cl, with a day-to-day variability much higher in Occasion 3 (around the 24-h trough) compared with the other occasions.

**Figure 1:**
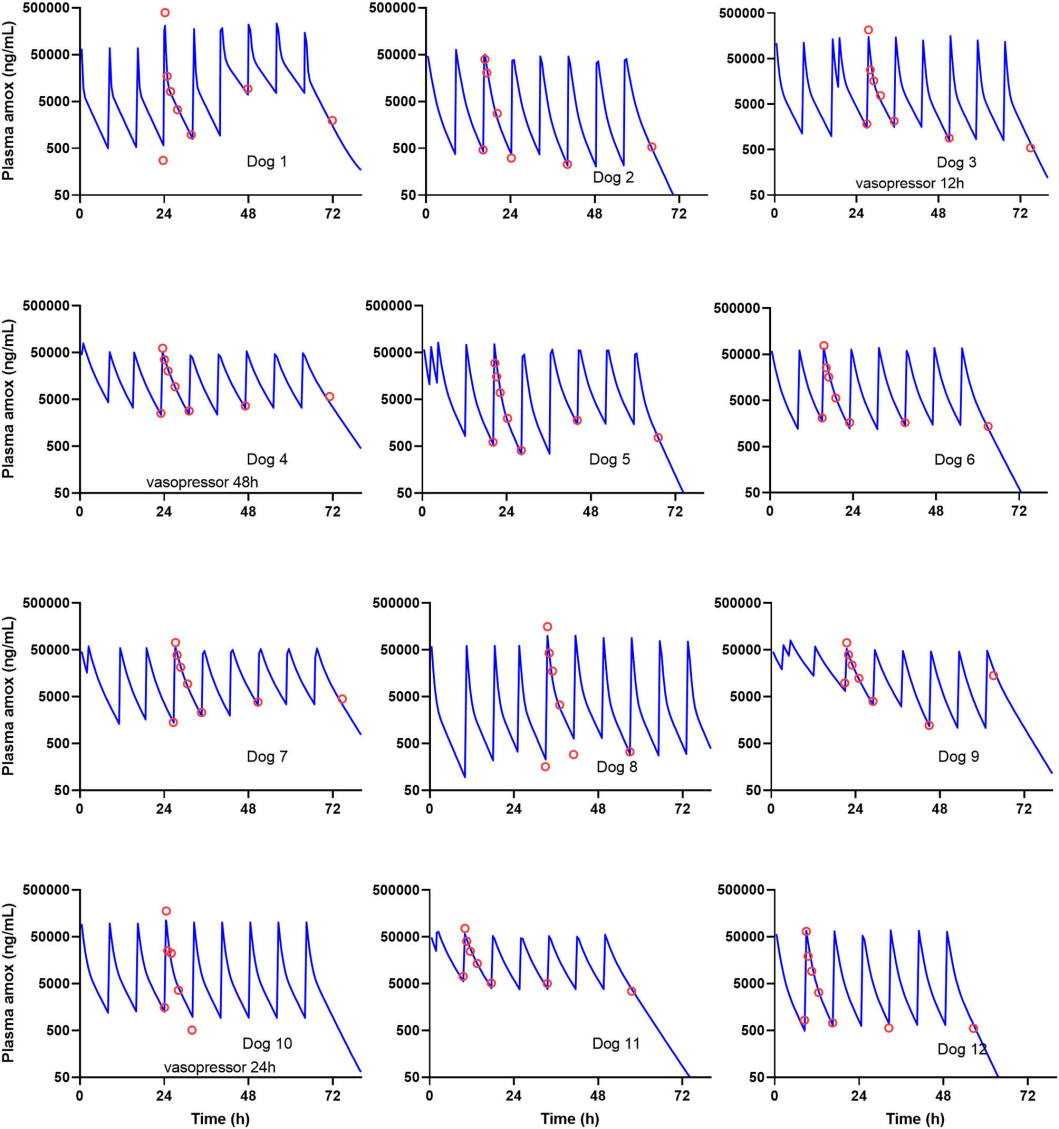
Observed (red circles) vs. predicted (blue lines) concentrations for the 12 sick clinical dogs. Time 0 h was the first recorded administration; the first plasma concentration was measured 16 to 24 h thereafter. Trough variability was observed and modeled including between-occasion variability (BOV) in the pharmacokinetic (PK) model. Individual prediction, from 0 to 96 h, was based on empirical Bayes estimates, i.e., *post-hoc* estimates of individual parameters.

The population parameter estimates (fixed and random effects) and their precision are summarized in Table 4. Amoxicillin did accumulate between doses in plasma of sick dogs. The individual fitting for sick dogs as obtained from the population model with *post-hoc* individual parameters is presented in Figure 1.

**Table 4:** Population pharmacokinetic estimates of amoxicillin in healthy and sick dogs.

**Parameter^**a**^**	**Estimate**	**Median bootstrap estimate**	**2.5th percentile from bootstrap^**b**^**	**97.5th percentile from bootstrap^**b**^**
Cl (L/kg/h)	0.336	0.332	0.293	0.384
V1 (L/kg)	0.173	0.182	0.119	0.212
Cl2 (L/kg/h)	0.861	0.796	0.717	1.007
V2 (L/kg)	0.174	0.172	0.166	0.193
Cl3 (L/kg/h)	0.0373	0.0368	0.0291	0.0515
V3 (L/kg)	0.0776	0.0754	0.0627	0.0915
θ (Health) on Cl	−0.829	−0.795	−0.975	−0.570
θ (Health) on Cl2	−9.946	−8.665	−11.989	−6.910
BSV Cl (CV%)	20.2	20.9		
BSV V1 (CV%)	60.2	67.4		
BSV Cl2 (CV%)	42.3	38.7		
BSV V2 (CV%)	9.2	16.0		
BOV on Cl (all but Occasion 3) (CV%)	18.07	47		
BOV on Cl (Occasion 3) (%CV)	42.14	19.4		
Proportional error healthy (CV %)	4.2%	4.1%	3.4%	5.1%
Proportional error sick (CV %)	35.9%	34.6%	25.2%	48.7%
Additive error healthy (ng/mL)	30.6	24.9	8.6	43.1
Additive error sick (ng/mL)	3.58	3.60	2.53	7.02

*^a^Cl, clearance; V1, central volume of distribution, Cl2 and Cl3, intercompartmental clearances; V2 and V3, peripheral volumes of distribution; these values are those of control dogs. θ, model parameters for covariate on Cl and Cl2. When θ_(HealthCl)_ = −0.829, this means that the plasma clearance in sick dogs is equal to that of a healthy dog times exp^(−0.829)^, i.e., 0.147 L/kg/h, or 43.6% of control value. For Cl2, a θ_(HealthCl2)_ of −9.946 means that V2 is negligible and no longer identifiable in sick dogs and that compartments 1 and 3 can describe the amoxicillin disposition in sick dogs; BSV, between-subject variability; BOV, between occasion variability of sick dogs (see text for Occasion description); CV, coefficient of variation.*

*^b^Non-parametric bootstrap of 30 successful runs for sick and healthy dogs*.

Covariate analysis identified that the total clearance in sick dogs was 0.147 L/kg/h, which corresponds to a 56% reduction compared with the clearance of healthy dogs (0.336 L/kg/h). Intercompartmental clearance was also decreased in sick dogs, to the extent where V2 was no longer identifiable in sick dogs, leaving compartments 1 and 3 to describe the amoxicillin disposition in sick dogs.

The individual fitting of the sick dogs, where BOV was included in the model, is presented in Figure 1. None of the clinical data covariates were significantly correlated with individual exposure, i.e., model derived AUC_0−64*h*_ (Spearman's R test).

**Figure 2:**
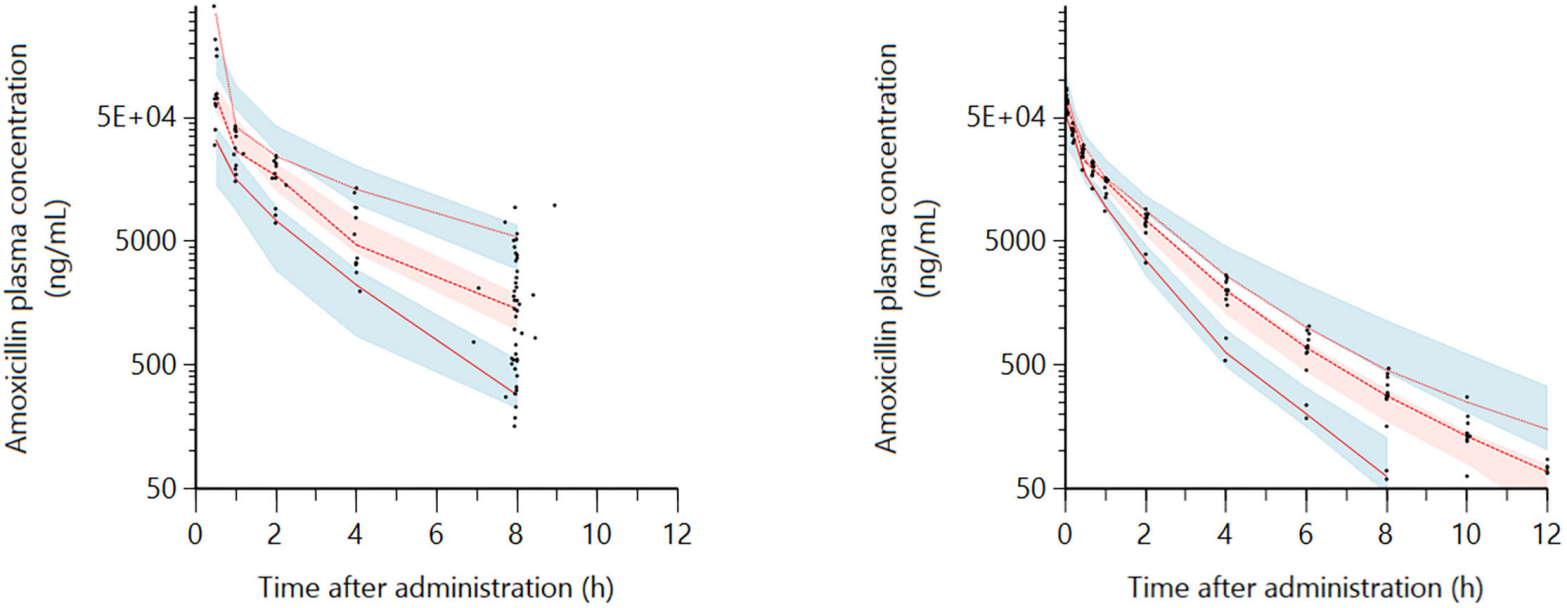
Visual Predictive Check (VPC) for the intravenous (IV) route obtained with 300 replicates of each animal in the dataset (sick dogs on the left plot and healthy dogs on the right plot). Red lines: observed quantiles (10th, 50th, and 90th). Black symbols: observed amoxicillin plasma concentration data (ng/mL). Blue and red shaded areas: the 90% confidence interval of predicted quantiles (10th, 50th, and 90th). The observed quantiles (10th, 50th, and 90th) are rather well-superimposed with the corresponding predictive check quantiles over the observed data. Theoretically, about 20% of data should be outside the plotted quantiles.

VPC plots are presented in Figure 2. The 10th, 50th, and 90th percentiles of the predicted concentrations follow closely those of the observed data, validating the model fit for both populations. Additional goodness-of-fit plots as well as model code are included in the Supplementary Material.

### Protein Binding

The protein binding determined by the ultrafiltration method was considered negligible. Indeed, in most samples from the sick dogs (concentration range 160 to 162,000 ng/mL) and in the sample from the healthy dog (at 150, 3,000, and 20,000 ng/mL), the free concentration was not different from total concentration, with the exception of a single sample from one sick dog with a value of protein binding of 24%.

### Monte Carlo Simulations

**Figure 3:**
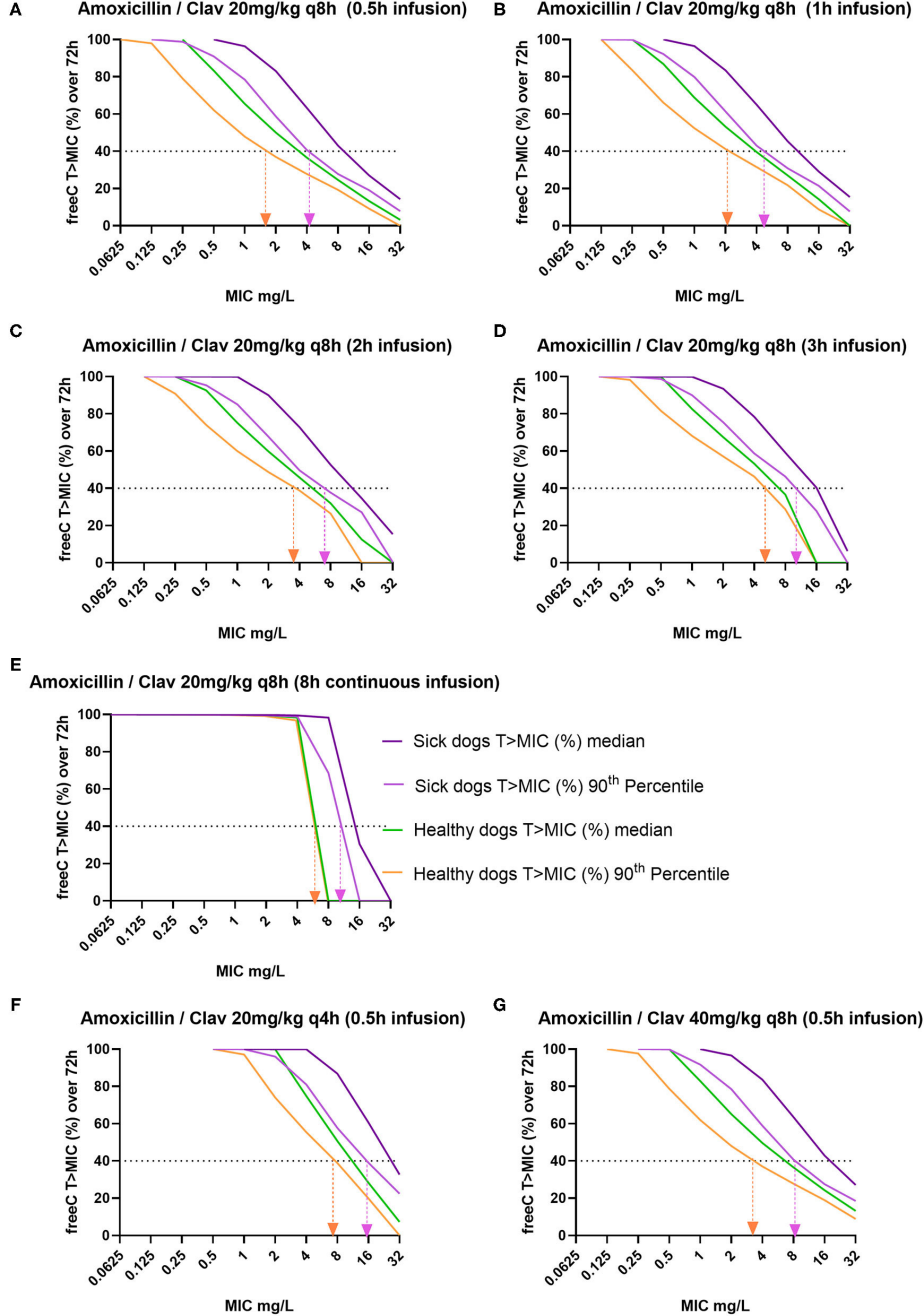
Percentage of time (within a 72-h dosing interval) for which free plasma concentrations exceeds minimum inhibitory concentration (MIC) (fT > MIC%) for the median and the 90th percentile of healthy and sick dogs (*n* = 1,000 simulations). Healthy dogs' percentiles, represented by green (50th) and orange lines (90th), were simulated from the ENVT population; sick dogs' percentiles, represented by dark (50th) and light (90th) purple, were simulated from the Royal Veterinary College (RVC) population. Vertical arrow projections indicate the pharmacokinetic/pharmacodynamic cutoff (PK/PD_CO_), with the lower bound of the MIC interval reported. The simulated dosage regimens of intravenous amoxicillin clavulanic acid were as follows: (i) with normal doses **(A)** 20 mg/kg (16.667 mg/kg of amoxicillin) every (q) 8 h, given in 0.5-h infusion; **(B)** 20 mg/kg q 8 h given in 1-h infusion, **(C)** 20 mg/kg q 8 h given in 2-h infusion, **(D)** 20 mg/kg q 8 h given in 3-h infusion, and **(E)** 20 mg/kg q 8 h, 8-h continuous infusion; and (ii) with increased doses **(F)** 20 mg/kg q 4 h given in 0.5-h infusion and **(G)** 40 mg/kg q 8 h given in 0.5-h infusion.

The percentage within a 72-h simulated treatment, for which free plasma concentrations exceed >MIC for the 50th (median) and 90th percentile of population individuals, is presented in Figure 3. The seven dosage regimens were evaluated for all possible MICs, ranging from 0.0625 to 32 mg/L. With the use of an AMC dose of 20 mg/kg administered over 30 min (standard regimen, Figure 3A), PK/PD_CO_ values were 1 and 4 mg/L for healthy and sick dogs, respectively. Higher PK/PD cutoff values could be reached by extending the length of the infusion to up to 3 h (Figure 3D), yielding PK/PD_CO_ of 4 and 8 mg/L for healthy and sick dogs, respectively. Achievement of PK/PD target with continuous infusion (Figure 3E) was similar as with 3-h extended infusion for a target of 40% of the dosing interval. However, administration by continuous rate infusion over 8 h remained superior to all other regimens for achieving free concentration exceeding MIC for 100% of the dosing interval, with a corresponding PK/PD_CO_ of 4 mg/L for both populations of dogs. When doubling the dose, the strategy of administering 20 mg/kg of AMC every 4 h was superior to administering 40 mg/kg every 8 h and allowed the coverage of MIC up to 16 mg/L in sick dogs.

## Discussion

Amoxicillin plasma concentration time curves from healthy beagle dogs and in critically ill dogs hospitalized in an ICU, following IV administration of AMC, were modeled in a unifying pharmacometric model. Clearance, distribution, and exposure to amoxicillin were profoundly affected by inflammation and infection. In our clinical multibreed population, 91% of dogs met SIRS criteria, and 83% had a confirmed source of infection or septic focus. In our control dogs, plasma clearance (0.336 L/kg/h) was similar to that previously published in healthy experimental dogs, which ranged between 0.204 and 0.270 L/kg/h (16, 22). In contrast, the plasma clearance of clinical dogs was greatly reduced by 56%, which means that, for a given dose, the internal exposure to amoxicillin in clinical dogs is more than doubled compared with that in control dogs. These results were not totally unexpected, as they are in line with observations in dogs injected experimentally with low and high doses of lipopolysaccharide (LPS) (14). These results may have important practical implications for the interpretation of CBPs for dogs hospitalized in an ICU. For a dose-dependent antimicrobial for which the PK/PD index is fAUC/MIC, halving the clearance would be equivalent to doubling the dose and targeting a CBP that would be increased by a single dilution factor. For a time-dependent AMD like amoxicillin, the situation is more complicated given the non-linearity between dose, form of internal exposure, and %fT > MIC (23). This difficulty can only be overcome with Monte Carlo simulations capable of predicting plasma concentrations profiles with their BSV for different administration scenarios, allowing to revisit the interpretation of the current CBPs.

In the presence of infection, in people, multiple factors associated with inflammation and therapeutic management have been described to contribute to underexposure more commonly than overexposure (10, 11, 24). Glomerular filtration rate (GFR) is probably the most significant covariate to impact on the clearance of beta-lactam antimicrobial in humans. Therapeutic drug monitoring (TDM) of beta-lactam concentration showed that 80% of the patients with renal dysfunction required a dose decrease and showed concentrations in excess of 10 times the MIC and commonly >100 mg/L (24). Although azotemic dogs are overexposed to ampicillin at common dosing regimen (15), and despite the relative overexposure of our amoxicillin dogs, we could not demonstrate a significant correlation between amoxicillin clearance and plasma creatinine in sick dogs. This was surprising as the control dogs from the current experimental study demonstrated a strong relationship between their plasma exo-iohexol and amoxicillin clearance (*R*^2^ = 0.96) (personal communication, Pelligand). We did not however measure iohexol clearance in the sick group due to the potential risk of contrast-induced nephropathy with iohexol in this population, and we cannot rule out that GFR measurement or markers of GFR could outperform plasma creatinine for predicting amoxicillin clearance (25). Sepsis-induced AKI is reported up to 12–13% in dogs, with abdominal sepsis being an important contributor to mortality (26–28), and we presently documented AKI in 2/12 dogs. Use of a lower dose of iohexol, measurement of cystatin C, or closed urine collection for the measurement of urinary creatinine clearance would be viable alternatives to plasma creatinine (29–31).

With regard to other covariates of interest, hypoalbuminemia is a common feature found in septic human and canine patients (32–36). In our study, 10/12 dogs had been reported to have hypoalbuminemia [albumin <26 g/L (R.I 26.3–38.2)] with 4/12 dogs presenting severe hypoalbuminemia below 16 g/L, but free amoxicillin concentrations were not directly measured in these dogs. There are misleading conceptions around the notion of drug binding to protein; the measurement of free concentration (from which the free fraction f_u_ is computed) is required for correct reasoning (37). Hypoalbuminemia, all things being equal, decreases the total drug plasma concentrations but does not change its free plasma concentration (hence it is f_u_ that is increased). This is because for all low extraction ratio drugs, regardless of route of administration, and for all drugs administered orally and eliminated primarily by the liver, total exposure is independent of protein binding (37, 38). Protein binding was negligible in our case but usually reported as low (~12%) for dogs (39). A higher value of 34% was reported with a microbiological assay, but a possible matrix effect flaws this result (40).

We observed high BOV in our population of sick dogs; this variability within the same individual was reflected by changes in trough concentrations along time. Our hypothesis is that as clinical condition improves, so does the renal clearance; and healthier, recovering dogs will have higher clearance and perfusion. This finding is important as TDM, together with population PK modeling, could be used as a tool to assess both individual exposure (and AUC/MIC or C_trough_/MIC as predictor of clinical cure) and an individual marker for GFR. While MIC measurement is becoming commonplace in diagnostic laboratories, several methods have been developed and validated for TDM of beta-lactam antimicrobials, but most use chromatographic separation coupled to ultraviolet or more sensitive mass spectrometry detection (41). Instrument cost, dosing expertise, and quality assurance and most importantly turnaround time (42) are limitations yet to be overcome.

According to the EUCAST rationale document, taking %fT > MIC of 30–40% and >90% target attainment rate, the most common IV dose used in people (500 mg every 8 h) results in an S breakpoint for systemic infection of 2 mg/L but could reach 8 mg/L with increased exposure (2 g every 6 h) (7). For veterinary medicine, standard IV doses (20 mg/kg every 8 h) yield a PK/PD_CO_ of 1 mg/L in healthy dogs and 4 mg/L in sick dogs (Figure 3A). Our Monte Carlo simulations showed that the highest PK/PD_CO_ values of 8 mg/L (*E. coli* ECOFF value) could be reached in sick dogs by extending the length of the infusion to up to 3 h (Figure 3D), still with 40% fT > MIC target. This is remarkable, as this dosing regimen could provide similar exposure to doubling the dose every 8 h (Figure 3G). Veterinary medicine lacks large clinical studies to corroborate these predictions, but a recent meta-analysis including 18 randomized controlled trials and 13 non-randomized trials demonstrated significantly lower overall mortality and improved clinical cure in people receiving constant or extended infusions of beta-lactams vs. intermittent bolus (43).

The (S) CBP reported for wild-type population of *E. coli* by the CLSI (8) for AMC is 0.25 mg/L for systemic infections (with a resistant CBP ≥ 1 mg/L). It refers to a dosage regimen of 11 mg/kg for amoxicillin with clavulanic acid administered orally every 12 h. Differences in CBP between organizations reflect not only species differences (man vs. dogs) in terms of PK and dosage regimen but also differences in (i) routes of administration (IV vs. oral) and (ii) selection of PK/PD target that is used in the computation of the PK/PD_CO_ (i.e., the value of the PK/PD index to achieve for ensuring the likelihood of a clinical cure). For amoxicillin, the PK/PD index selected is nearly always the %fT > MIC, but the target values to achieve might be different, ranging from 30–40% (7, 10) to 90–100% of the dosing interval (8) or even requiring to achieve plasma concentration several times above the MIC during the whole dosage interval (44). This target selection may explain on its own the CBP difference, which varies by several dilutions because %fT > MIC is very sensitive to the shape of the plasma concentration curve, which can even lead to jump discontinuities, as explained by Toutain et al. (23).

There are several limitations in our study. First, although we were able to identify the differences in Cl, exposure, and variability within the sick dogs compared with the healthy group, the study was underpowered to assess the covariate effects on the model. A study limitation was the greater interindividual variability observed in PK parameters in the sick group partly explained also by the use of dogs of different breeds compared only with female beagles. We aimed to generate hypotheses for exploring the effect of different disease and treatment covariates on the PK of AMC. Secondly, we have not analyzed the PK population for clavulanic acid for this study. Despite its essential action in increasing the antimicrobial effect of amoxicillin, it has no antimicrobial effect on its own. We will report further information regarding clavulanic acid PKs in the near future. Thirdly, the free concentration should ideally have been measured from plasma samples of the dogs involved in the PK study, but this was not technically possible at the time. Furthermore, the reference method (equilibrium dialysis) should be used to confirm this finding (45). Finally, no MIC values were measured prospectively, and we deliberately compared the PK/PD_CO_ with the highest MIC of the wild-type distribution of *E. coli* (8 mg/L), as a worst-case scenario. For any bacterial species with a lower ECOFF and values within the MIC range, we would predict better exposure and outcome (for example, methicillin-sensitive *Staphylococcus* or *Pasteurella*).

In conclusion, the PK of IV amoxicillin was profoundly different in critically ill dogs compared with normal dogs, with much higher interindividual variability and lower systemic and intercompartmental clearances. As seen in the present case, a critical reflection upon reliance on a single breakpoint common to all routes and patient strata is warranted. Reporting CBP S ≤ 0.25 and R ≥1 mg/L precludes the selection of IV AMC for those treatable bacteria with MICs from 1 to 8 mg/L, to escalate to a more critical antimicrobial. In the era of the prudent use of antimicrobials and personalized medicine, a reflection merits to be initiated in veterinary medicine so that diagnostic laboratories take into account and integrate in their reports all the information available to allow the most judicious use of antimicrobials with a possible Bayesian approach to inform a dose adjustment (46).

## Data Availability Statement

The raw data supporting the conclusions of this article will be made available by the authors, without undue reservation.

## Ethics Statement

The animal study was reviewed and approved by two institutions. Both studies were performed under the European Directive 2010/63/EU on the protection of animals used for scientific purposes. For the RVC dogs, the study protocol was approved by the Animal Welfare and Ethical Review Board. The satellite study was authorized by the French Ministry of Research and approved by the Ethical Committee for Pharmacology Toxicology Midi-Pyrénées (n°86). Written informed consent was obtained from the owners for the participation of their animals in this study.

## Author Contributions

MV conducted the experiments, curated the data, analyzed and interpreted the results, and co-wrote the manuscript. SC contributed to the study design, organized the experiments, analyzed and interpreted the results, and co-wrote the manuscript. MC carried out the amoxicillin measurement and co-wrote the manuscript. MD contributed to the study design, organized the sample import and analysis, and edited the manuscript. BR contributed to the study design, conducted the experiments, curated the data, and co-wrote the manuscript. AB-M contributed to the study design/hypothesis generation and edited the manuscript. P-LT contributed to study design/hypothesis generation, interpreted and analyzed the results of PK/PD modeling, and co-wrote the manuscript. LP designed and coordinated the study, organized the experiments, carried out PK/PD modeling, and co-wrote the manuscript. All authors contributed to the article and approved the submitted version.

## Funding

The authors are thankful for the Scil EVECC Research Grant that supported part of the study (RVC grant 5082) and the in-kind contribution from ENVT and the Ghent analytical laboratory. This article is based upon work from COST Action 18217, publication fees were supported by COST (European Cooperation in Science and Technology; www.cost.eu), a funding agency for research and innovation networks.

## Conflict of Interest

The authors declare that the research was conducted in the absence of any commercial or financial relationships that could be construed as a potential conflict of interest.

## Publisher's Note

All claims expressed in this article are solely those of the authors and do not necessarily represent those of their affiliated organizations, or those of the publisher, the editors and the reviewers. Any product that may be evaluated in this article, or claim that may be made by its manufacturer, is not guaranteed or endorsed by the publisher.
