# Supplementary material for: Population Pharmacokinetics of Intravenous Amoxicillin Combined With Clavulanic Acid in Healthy and Critically Ill Dogs
Source: Front Vet Sci. 2021 Nov 15;8:770202. doi: 10.3389/fvets.2021.770202 (PMC8636140; doi:10.3389/fvets.2021.770202)

Supplementary Material

1 Supplementary Figures

Supplementary Figure 1: Plot of the dependent variable (DV, i.e. of observed plasma amoxicillin concentrations in ng/mL) versus individual predicted plasma amoxicillin values (IPRED). Individual predictions are obtained by setting random effects to the 'post hoc' or empirical Bayesian estimate of the random effects for the individual from which the plasma concentration observation was made. Thus, the plot shows observed vs fitted values of the model function. Ideally, they should fall close to the line of unity y=x. Top two figures on arithmetic scale and bottom two figures on logarithmic scale (clinical dogs on the left, healthy dogs on the right).

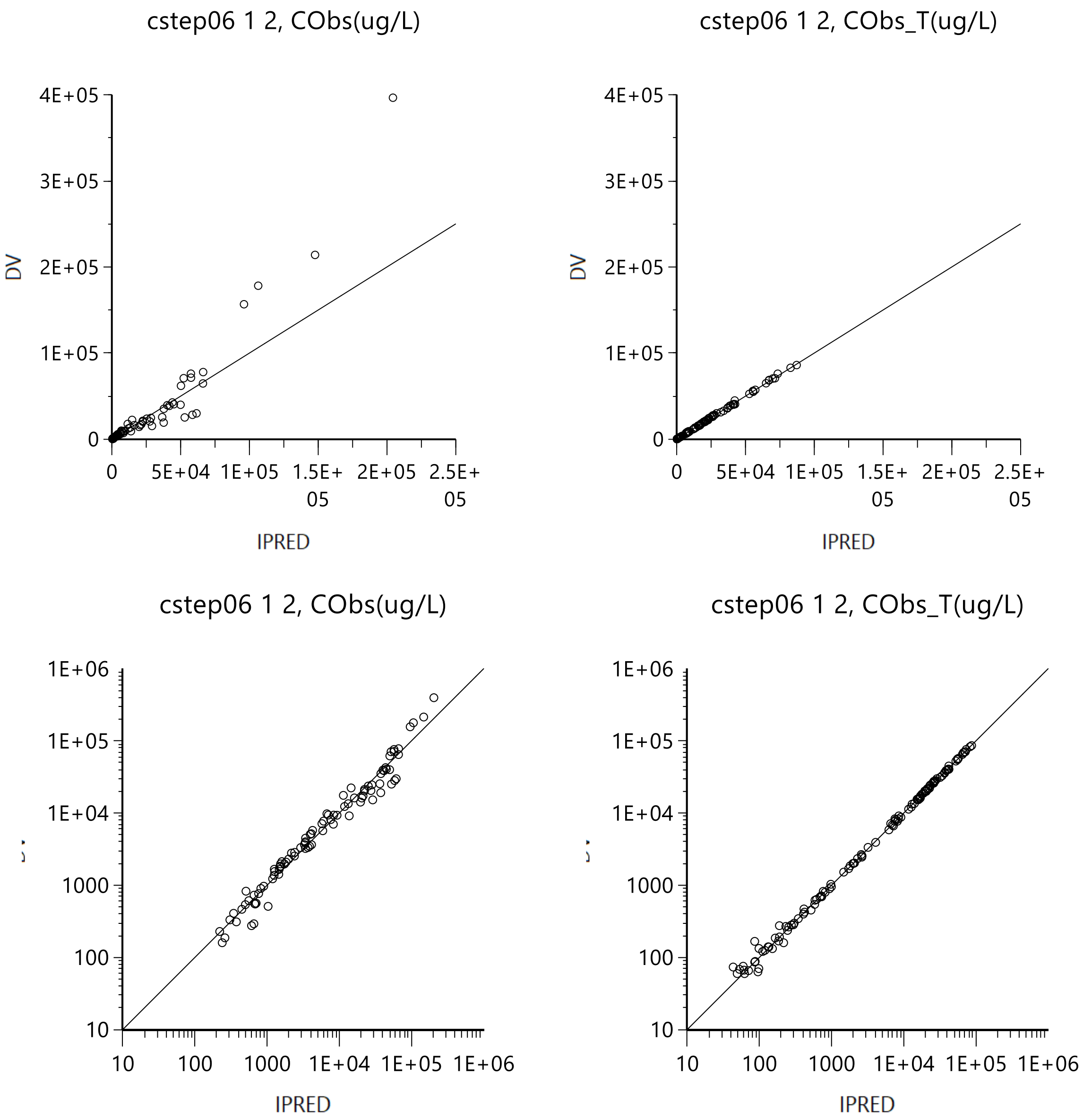

Supplementary Figure 2: Plot of the dependent variable (DV, i.e. of plasma amoxicillin concentrations ng/mL) versus population predicted plasma amoxicillin concentrations (PRED) (no random component). The plot shows observed vs. fitted values of the model function. Ideally, they should fall close to the line of unity y=x. Top two figures on arithmetic scale and bottom two figures on logarithmic scale (clinical dogs on the left, healthy dogs on the right).

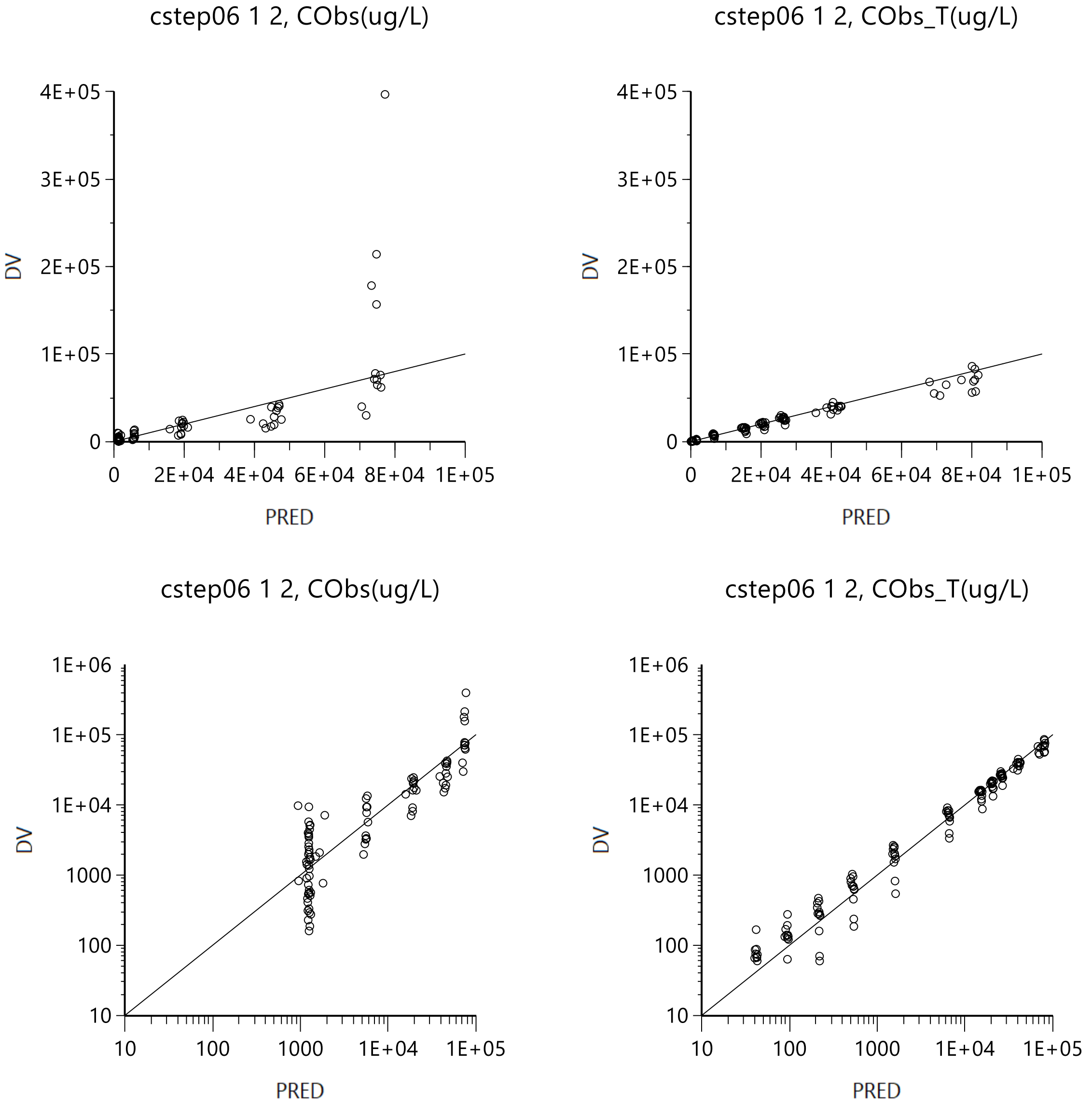

Supplementary Figure 3: Histogram plot of the conditional weighted residual values (CWRES). Values of CWRES should be approximately distributed around 0, N(0,1) and hence concentrated. Clinical dogs on the left, healthy dogs on the right. The figure corresponding to the clinical dogs is on the left, the figure for healthy dogs is on the right.

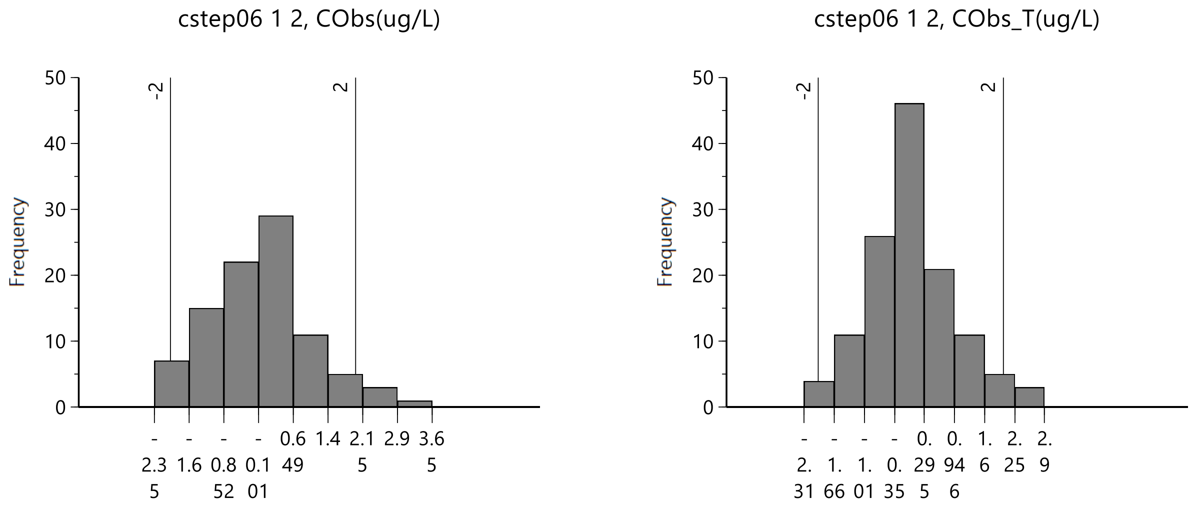

Supplementary Figure 4: Plot of Conditional Weighted Residuals (CWRES), a proposed replacement for the classical WRES (weighted residuals) goodness of fit statistic, against Time after Dose (TAD, in h). Clinical dogs on the left, healthy dogs on the right. Values of CWRES should be approximately normally distributed around 0, N(0,1) and hence concentrated between y=-2 and y=+2. Values significantly above 3 or below -3 are suspect and may indicate a lack of fit and/or model misspecification. The figure corresponding to the clinical dogs is on the left, the figure for healthy dogs is on the right.

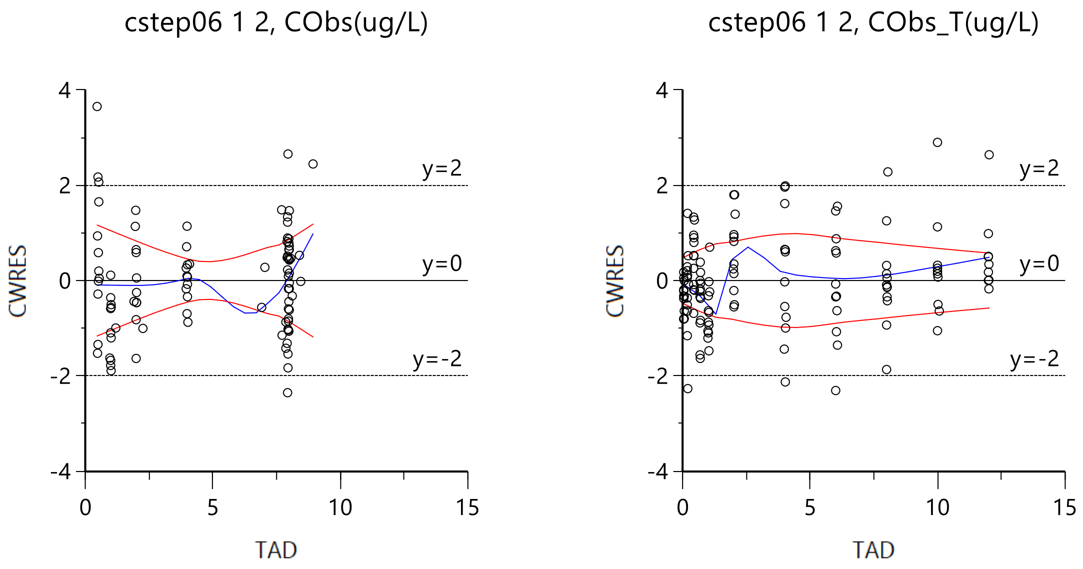

Supplementary Figure 5: Plot of Conditional Weighted Residual (CWRES), against the population predictions (PRED in ng/mL, i.e. the predictions obtained by setting the random effect values to zero) used for the x axis. Clinical dogs on the left, healthy dogs on the right. Values of CWRES should be approximately normally distributed around 0, N(0,1) and hence concentrated between y=-2 and y=+2. Values significantly above 3 or below -3 are suspect and may indicate a lack of fit and/or model misspecification. The figure corresponding to the clinical dogs is on the left, the figure for healthy dogs is on the right.

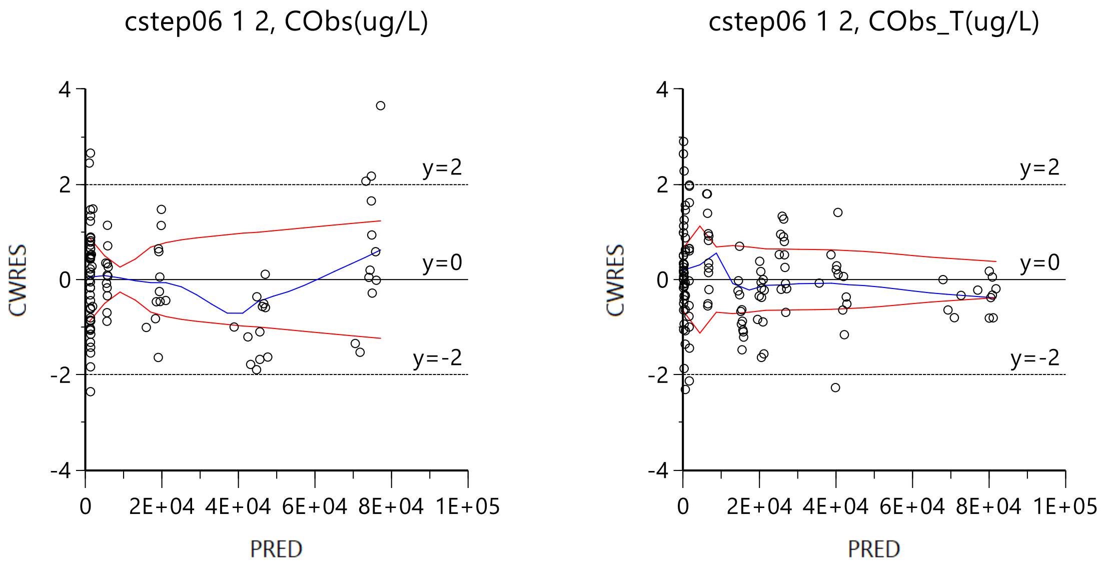

Supplement: Supplementary file 1 [file Data_Sheet_1.pdf]
